# Covalent Attachment of Active Enzymes to Upconversion Phosphors Allows Ratiometric Detection of Substrates

**DOI:** 10.1002/chem.202001974

**Published:** 2020-10-16

**Authors:** Letitia Burgess, Hannah Wilson, Alex R. Jones, Peter Harvey, Louise S. Natrajan, Sam Hay

**Affiliations:** ^1^ Department of Chemistry School of Natural Sciences The University of Manchester Oxford Road Manchester M13 9PL United Kingdom; ^2^ Manchester Institute of Biotechnology The University of Manchester 131 Princess Street Manchester M1 7DN United Kingdom; ^3^ Photon Science Institute The University of Manchester Oxford Road Manchester M13 9PL United Kingdom; ^4^ Biometrology, Chemical and Biological Sciences, National Physical Laboratory Hampton Road Teddington, Middlesex TW11 0LW United Kingdom; ^5^ School of Medicine The University of Nottingham University Park Nottingham NG7 2RD United Kingdom

**Keywords:** biosensors, energy transfer, enzymes, lanthanides, upconversion

## Abstract

Upconverting phosphors (UCPs) convert multiple low energy photons into higher energy emission via the process of photon upconversion and offer an attractive alternative to organic fluorophores for use as luminescent probes. Here, UCPs were capped with functionalized silica in order to provide a surface to covalently conjugate proteins with surface‐accessible cysteines. Variants of green fluorescent protein (GFP) and the flavoenzyme pentaerythritol tetranitrate reductase (PETNR) were then attached via maleimide‐thiol coupling in order to allow energy transfer from the UCP to the GFP or flavin cofactor of PETNR, respectively. PETNR retains its activity when coupled to the UCPs, which allows reversible detection of enzyme substrates via ratiometric sensing of the enzyme redox state.

## Graphical abstract

**Variants of GFP** and the flavoenzyme PETNR were attached to upconverting phosphors (UCPs) via maleimide‐thiol coupling in order to allow apparent energy transfer from the UCP to the cofactors of GFP or PETNR. PETNR retains its activity when coupled to the UCPs, which allows reversible detection of enzyme substrates via ratiometric sensing of the enzyme redox state.

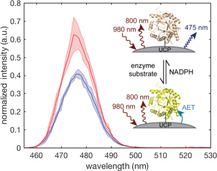

Upconverting phosphors (UCPs) have emerged as an important and versatile class of nanoparticles, with applications including memory storage, anti‐counterfeiting measures, theranostics, and optical imaging.^[1]^ Upconversion (UC) involves the sequential absorption of two or more lower energy photons that results in the emission of light of higher energy. Typically, near‐infrared (NIR) excitation of UCPs results in visible luminescence.^[2]^ While UC has recently been shown in small molecule complexes,^[3]^ the most common systems are based on Yb^III^→Er^III^ or Yb^III^→Tm^III^ rare‐earth ion pairs doped into an inert matrix (e.g., NaYF_4_, Gd_2_O_2_S, etc.),^[2]^ and more recently Nd^III^ ions have been used in place of Yb^III^ to enable excitation at 808 nm, where water and biological tissue absorb less strongly. ^[ 1f]^

UCPs have a number of potential advantages over traditional fluorophores, including: a large anti‐Stokes shift; an associated lack of auto‐fluorescence in biological media due to their NIR excitation; negligible photobleaching; no photo‐blinking and generally low toxicity.^[4]^ In addition, due to the contracted nature of the lanthanide(III) f orbitals, emission wavelengths in Ln^III^‐based UCPs are generally insensitive to particle size and environment and their long (typically μs‐ms) lifetimes enable time‐gated spectroscopic measurements to be employed if required.^[5]^ Due to these favorable properties, UCPs have been proposed for use in a range of sensing and imaging applications, from heavy metal detection to image‐guided photodynamic therapy.^[6]^ An as‐yet untapped application is the covalent attachment of active biomolecules to UCPs where the biomolecule can act as an acceptor for the UC emission, although UCP biomolecule conjugates (including DNA) have been developed where the biomolecule can be electrostatically surface bound.^[7]^ The UCP would then act as a robust luminescent reporter of, for example, the redox or ligand‐bound state of the UC acceptor (Scheme 1, inset). Enzyme activity is typically monitored by following changes in concentration of the substrate/product, or by directly following the enzyme in the case of single‐turnover experiments. Fluorescence detection allows experiments to be performed at lower concentrations, but typically relies on high‐energy (single photon) excitation, which can lead to photobleaching. The use of UCP‐conjugates, and intrinsic low energy excitation, may eventually allow such experiments to be carried out in media with high‐scattering, significant auto‐fluorescence, and/or where increased sample penetration depth is required (e.g. biological tissue). These factors make UCP‐conjugates more amenable for use in biological and environmental sensing applications than by monitoring enzymes or substrates directly.

**Scheme 1:**
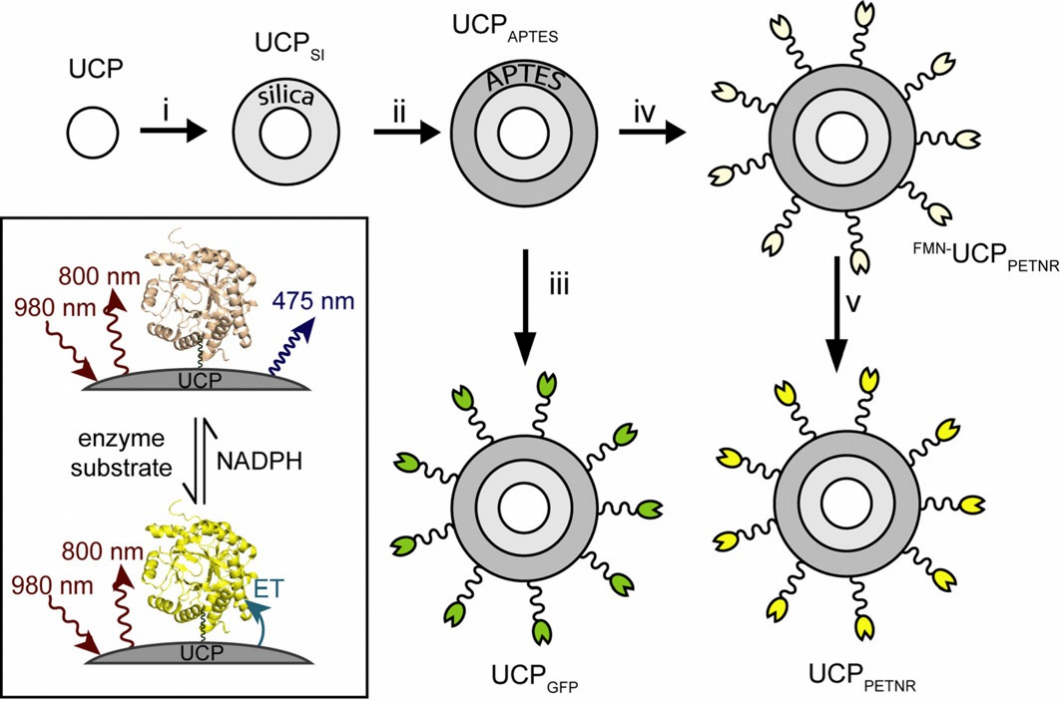
Simplified representation of overall synthetic scheme; i) Igepal CO‐520, NH_4_OH, TEOS, cyclohexane; ii) APTES, cyclohexane; iii) Sulfo‐SMCC, GFP, PBS; iv) Sulfo‐SMCC, PETNR, PBS; v) KBr, FMN, PBS; see Supporting Information for additional experimental details. Inset shows “on–off” apparent energy transfer (AET) concept with PETNR on the surface of UCPs.

We have previously demonstrated significant diffusion‐controlled quenching of UCP upconversion emission by the oxidized flavin cofactor of the enzymes pentaerythritol tetranitrate reductase (PETNR)^[8]^ and glucose oxidase,^[9]^ as well as to vitamin B_12_, and the heme cofactor of cytochrome c.^[9]^ This quenching could be a result of an emission‐reabsorption (secondary inner filter effect) process and/or direct energy transfer from UCP to chromophore if their separation (Förster distance) is sufficiently short (i.e. quenching via FRET or LRET). As the photophysical mechanism of UCP quenching is not the focus of the present study, we will collectively refer to the UCP quenching process as apparent energy transfer (AET). Previous studies have also demonstrated covalent attachment of biomolecules to UCP surfaces,^[10]^ but have not exploited AET from the UCP as a spectroscopic probe. The closest example used AET from UCPs to glucose oxidase immobilized on poly(acrylamide) for flow‐based applications.^[11]^ Here, we have now created covalent UCP‐protein/enzyme conjugates that undergo intra‐system AET from the UCP to the protein cofactor while suspended in aqueous solution. We chose two exemplar proteins with different intrinsic chromophores: enhanced green fluorescent protein (GFP) and PETNR. The methodology should be applicable to any protein that possesses a native or engineered surface‐exposed cysteine residue, so can be adopted by those currently using thiol or maleimide‐based organic fluorescent probes.^[12]^

GFP contains the chromophore *p*‐hydroxybenzylidene‐2,3‐dimethylimidazolidine (HBDI),^[13]^ which has absorption maxima at 395 and 475 nm (Figure 1). The latter absorption band overlaps with the 475 nm emission band (^1^G_4_→^3^H_6_ transition) of Tm^III^‐doped UCPs, and therefore has the potential for efficient AET from UCP to GFP (Figure 1 A). Enhanced GFP contains two cysteine residues with one, C48, partially solvent‐exposed (Figure S1). Initially, GFP was covalently attached to the surface of maleimide‐capped ytterbium(III)–thulium(III) doped gadolinium oxysulfide UCPs (Gd_2_SO_2_:Yb:Tm, PTIR‐475) via direct maleimide‐thiol chemistry. However, as the UC emissive process is notoriously capricious and easy to quench, we found that any form of direct surface modification with maleimide‐containing groups led to loss of UC. Therefore, we first coated the UCPs in a silica layer. This layer of silica provides multiple benefits: protection of the UCP surface against quenching processes; fairly robust biocompatibility and the ability to functionalize further with ease.

**Figure 1:**
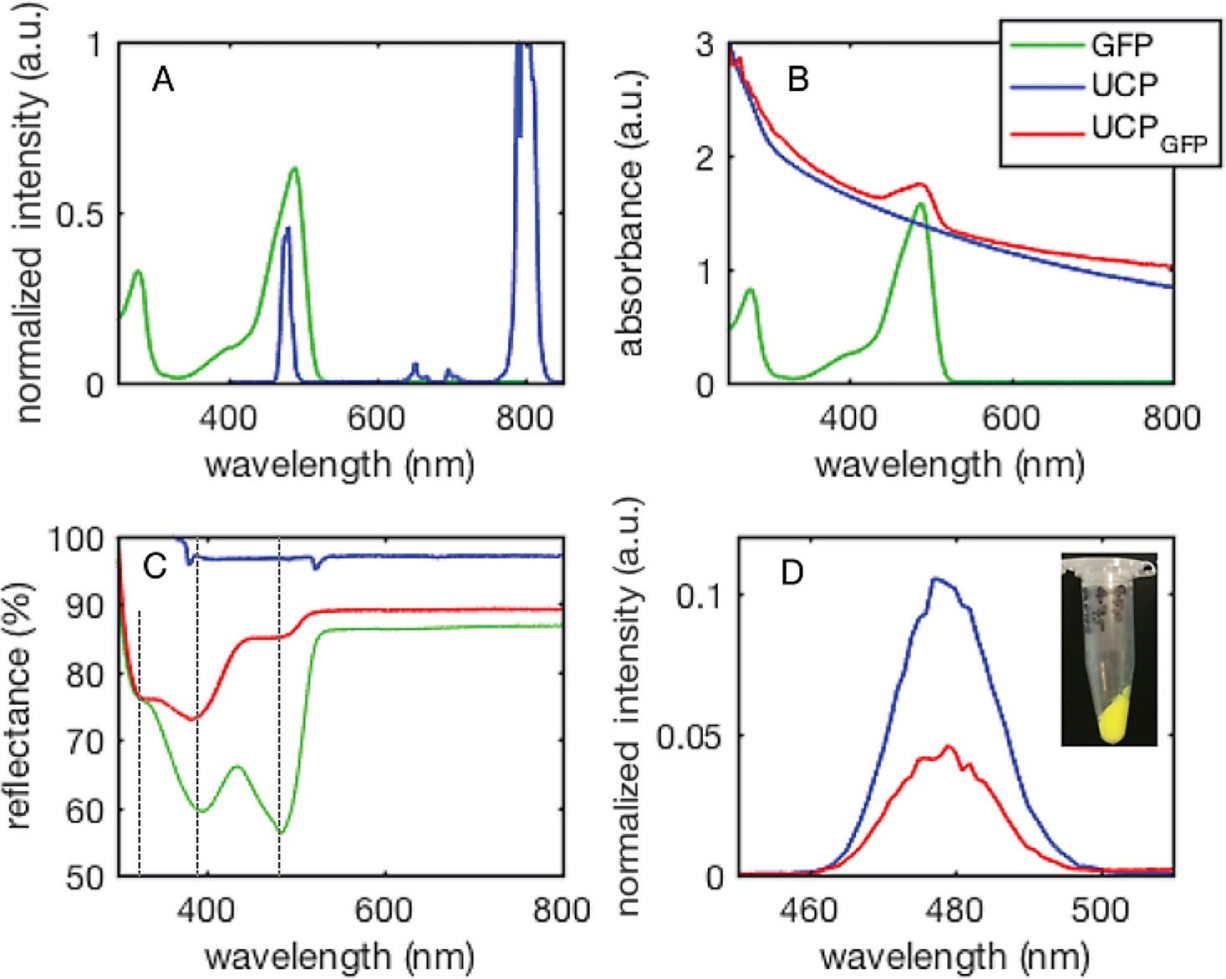
(A) Spectral overlap of GFP absorption (green) with UCP emission (blue). (B) Solution UV/Vis absorption spectra of GFP, UCP, and UCP_GFP_ in PBS. (C) Solid‐state UV/Vis reflectance spectra of GFP, UCP, and UCP_GFP_ separately drop‐cast and dried between two glass slides, with UCP_GFP_ displaying distinct GFP bands. (D) Upconversion emission spectra of UCP and UCP_GFP_ (*λ*_ex_=980 nm) in PBS. Bands are normalized to the 800 nm UC emission intensity with full spectra shown in Figure S3. (Inset) Photograph of UCP_GFP_ showing distinct luminous yellow‐green coloration from conjugated GFP. Universal legend for all panels: green=GFP, blue=UCP (PTIR‐475), red=UCP_GFP_.

PTIR‐475 UCPs were capped with silica using a reverse microemulsion synthesis, with IGEPAL® CO‐520 used to stabilize the procedure during the polymerization of tetraethyl orthosilicate.^[14]^ Then, (3‐aminopropyl)triethoxysilane (APTES) was added to the reaction mixture. APTES combines with the silica coating to present an accessible surface layer of primary amines, which can be modified relatively easily using *N*‐hydroxysuccinimide (NHS) esters. In order to covalently couple protein cysteines to the amine‐coated UCPs, sulfosuccinimidyl 4‐(*N*‐maleimidomethyl)cyclohexane‐1‐carboxylate (sulfo‐SMCC) was employed as a linker. This linker contains both an NHS‐ester and a maleimide and was first allowed to couple (via maleimide‐cysteine conjugation) to the protein before the addition of the APTES‐coated UCPs. The reaction mixture was gently agitated under mild conditions to allow the coupling to progress and after each stage of this multi‐step procedure the UCPs were centrifuged and washed several times to remove unreacted reagents; the overall synthetic Scheme is summarized in Scheme 1. The final particles, UCP_GFP_, were isolated as a luminous yellow‐green powder (Figure 1 D, inset). The average sizes of the unmodified UCP and surface modified UCP_APTES_ UCP_GFP_ particles were determined by dynamic light scattering (DLS) and transmission electron microscopy (TEM) measurements and are collated in Figures S9 and Table S1 in the Supporting Information. Averaged TEM measurements give particle sizes of 765 nm (UCP), 809 nm (UCP_APTES_) and 878 nm (UCP_GFP_).

UV‐visible absorption spectra were recorded from UCP_GFP_ particles suspended in phosphate buffered saline (PBS). While the particles cause a significant amount of scattering, a distinct peak is observed around 480 nm (Figure 1 B), characteristic of GFP absorption and this band is not observed in the unconjugated UCPs. Likewise, the solid‐state UV‐vis reflectance spectrum of UCP_GFP_ (Figure 1 C) also shows the characteristic 395 and 475 nm bands arising from GFP. The relative intensity of these bands differs to the those of GFP in solution, likely due to scattering from UCP_GFP_. Direct excitation of the GFP fluorophore at 475 nm gives rise to fluorescence emission at 530 nm, characteristic of GFP (Figure S2). Excitation of the particles with 980 nm light leads to UCP emission bands at 475, 650, and 800 nm, corresponding to the Tm^III^ transitions of ^1^G_4_→^3^H_6_, ^1^G_4_→^3^F_4_, and ^3^H_4_→^3^H_6_, respectively.^[15]^ Comparison of the UC emission from UCP_GFP_ relative to the APTES‐coated UCP showed a ≈60 % reduction in emission intensity of the 475 nm band, when normalized to the 800 nm peak (^3^H_4_→^3^H_6_ transition; Figures 1 D and S3). This decrease in emission is consistent with AET from the UCP to GFP, but no emission from GFP at 530 nm was observed, even at long accumulation times,^[8]^ suggesting that fluorescence from those GFP moieties acting as AET acceptors is efficiently quenched; this is likely to be due in part to a considerable reduction of the GFP quantum yield. Unfortunately, as previously observed, we were not able to infer any energy transfer from upconverted emission lifetime measurements.[^8^, ^9^] Using UV‐visible spectroscopy (Figure 1 B), we estimate 3.9 nanomoles of GFP are conjugated to 1 mg of UCP, resulting in a working concentration of 3.9 μm GFP in the 1 mg mL^−1^ UCP solutions (see Supporting Information).

If AET occurs by FRET or LRET, then incomplete quenching of UCP emission at 475 nm may be due to inefficient energy transfer from Tm^III^ sites distant from the UCP surface and/or due to emission from a small population of unreacted UCPs (unobserved by TEM analysis). UCP conjugates were also prepared with synthesized nanoparticles of smaller diameter of ≈20 nm (^mal^UCP, see the Supporting Information), which are small enough for FRET or LRET to occur. Similar AET behavior was observed (Figure S4), but the smaller particles required a non‐ideal, much higher power laser sources to generate comparable UC emission (≈1 W vs. 45 mW). Consequently, the commercial PTIR‐475 microparticles were used for the remainder of the current study.

Following the successful generation of UCP_GFP_, a similar synthetic route was used to conjugate PETNR to the UCPs. PETNR is an NAD(P)H [reduced nicotinamide adenine dinucleotide (phosphate)]‐dependent enzyme,^[16]^ which possesses a native surface‐accessible cysteine (Cys222; Figure S1) that is reactive towards thiol and maleimide derivatives of organic fluorophores.^[17]^ Like GFP, the 465 nm absorption band of the oxidized FMN cofactor of PETNR has good spectral overlap with the UCP Tm^III^ emission band at 475 nm.[^8^, ^9^] Upon reduction of PETNR by NAD(P)H, the 465 nm FMN absorption is lost, largely abolishing AET from the UCP, leading to an increase in UC emission from the UCP at 475 nm. Comparing intensity ratios with the 800 nm emission of the UCPs thereby provides a ratiometric description of the redox state of the FMN bound to PETNR.^[8]^ During the coupling procedure, much of the relatively weakly‐bound (non‐covalent) FMN cofactor disassociates from the enzyme. The FMN can be reincorporated into the apoenzyme,^[18]^ however, by soaking the apoenzyme‐conjugated UCP‐system (UCP_apo‐PETNR_) in a solution containing an excess of FMN and 1 m KBr to assist in FMN binding.^[18]^ After 24 hours of gentle agitation at 4 °C, the resulting particles, UCP_PETNR_, were isolated by centrifugation and repeatedly washed until FMN was no longer present in the supernatant (Figure S5). Throughout this procedure the color of the UCPs progressed from white (UCP_APTES_), to straw‐yellow (UCP_apo‐PETNR_), to a characteristic deep yellow/orange in UCP_PETNR_ (Figure 2, lower panel inset). Characterization by FTIR, TEM and DLS is shown in Figures S6–S9 and Table S1. These data show the presence of silica (capping) and organic matter (protein) in the samples and that the UCP_apo‐PETNR_ and UCP_PETNR_) particles have average sizes of 990 and 939 nm.

**Figure 2:**
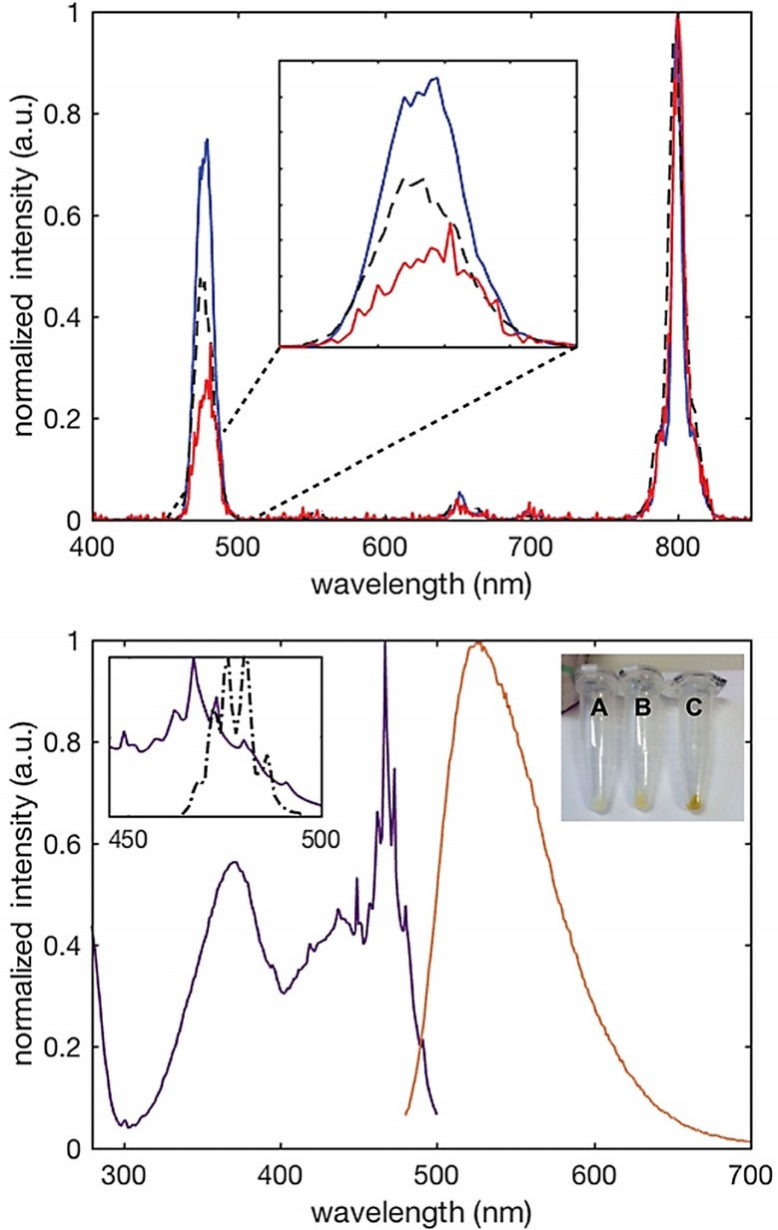
Upper panel) UC emission spectra of UCP_APTES_ (blue), UCP_apo‐PETNR_ (dashed black), and UCP_PETNR_ (red). All samples were in PBS, *λ*_ex_=980 nm. Inset shows expansion of 475 nm emission band. Lower panel) Direct excitation (violet, *λ*_em_=530 nm) and emission (orange, *λ*_ex_=448 nm) spectra of PETNR in UCP_PETNR_. Top left inset shows overlap of UCP_PETNR_ excitation spectrum with the resolved emission spectrum of UCP (black dashed; *λ*_ex_=980 nm). Top right inset shows the distinctive color change from UCP_APTES_ (A) to UCP_apo‐PETNR_ (B) and UCP_PETNR_ (C).

The UC emission spectra of UCP_APTES_, UCP_apo‐PETNR_ and UCP_PETNR_ are shown in Figure 2. Again, they all show the typical Tm^III^ emission at 475, 650, and 800 nm and were normalized to the 800 nm peak for comparison. There is a ≈60 % quenching of the 475 nm UC emission in UCP_PETNR_, consistent with AET from the UCP to the FMN cofactor in PETNR. Some quenching is also observed in UCP_apo‐PETNR_, likely due to low levels of bound FMN in this sample and/or some quenching of the UCP by e.g., vibrational relaxation due to the presence of the apoprotein. There has been some contention as to the exact nature of AET in UCP systems, with some evidence that it is highly dependent on the nature of the size and lattice of the UCP donor and the distance of emitter ions to the acceptor.^[19]^ The data here show the addition of the FMN cofactor to UCP_apo‐PETNR_ leads to significant quenching of the UC emission. This is consistent with quenching by AET to an acceptor chromophore with good spectral overlap with the UCP emission band(s), so this approach should be applicable to other suitable chromophores.

Direct excitation of the FMN in UCP_PETNR_ at 448 nm shows characteristic flavin emission at ≈530 nm (Figure 2), further indicating successful functionalization of the UCPs. The companion excitation spectrum shows the expected FMN excitation superimposed with fine structure, which is due to Tm^III^ emission from the UCPs at this wavelength (Figure 2, lower panel inset).

While these data collectively suggest we have successfully conjugated PETNR to the surface of UCPs, in order to be useful as model biosensor, the enzyme needs to retain its catalytic activity when bound to a UCP. Consequently, the steady‐state kinetics of UCP_PETNR_ were assessed. The simplified 2‐step reaction of PETNR is shown in Equations 1 and 2 (Simplified reaction Scheme for PETNR. *k*_RHR_ and *k*_OHR_ describe the reductive and oxidative half reactions, respectively, and S is an oxidative substrate such as ketoisophorone. Note that PENTR_ox_ can also be reduced with sodium dithionite) and kinetic data are shown in Figure 3.

(1) $${PETNR}_{ox}+NAD\left(P\right)H\;\overset{k_{RHR}}{\underset{}{\to }}{PETNR}_{red}+{NAD\left(P\right)}^{+}$$

(2) $${PETNR}_{red}+S+H^{+}\overset{k_{OHR}}{\underset{}{\to }}{PETNR}_{ox}+S-H_{2}$$

**Figure 3:**
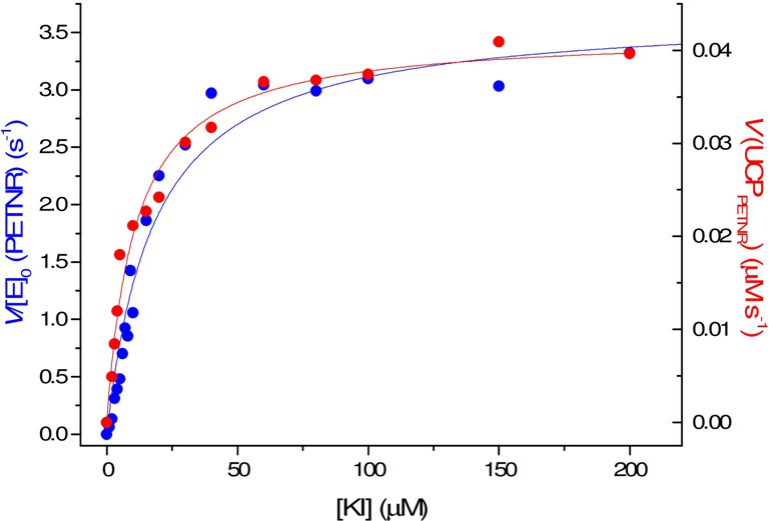
Michaelis–Menten plots for the reaction of PETNR (blue) and 1 mg mL^−1^ UCP_PETNR_ (red) with 100 μm NADPH and varying substrate, KI. These data are fitted to Equation (1) (solid lines).

As reduced PETNR will oxidize under ambient aerobic conditions the following experiments were performed under anaerobic conditions (N_2_ atmosphere) at room temperature. We found that PETNR is still active when bound to the UCPs and both PETNR and UCP_PETNR_ show typical “Michaelis‐Menten” behavior with the oxidizing substrate ketoisophorone (KI) when NADPH consumption is measured. These data were fitted to the Michaelis‐Menten equation [Eq. 3]:

(3) $$V_{\mathrm{obs}}=V_{\max\;}\left[S\right]/\left(K_{m}+\left[S\right]\right)\;\;V_{\max\;}=\;k_{\mathrm{cat}}/{\left[E\right]}_{0}$$

giving *K*_m_=18.1±2.4 μm and *k*_cat_=3.68±0.15 s^−1^ for PETNR in solution and *K*_m_=10.9±1.2 μm and *V*_max_=0.042±0.001 μm s^−1^ per mg mL^−1^ UCP_PETNR_ (Figure 3). Determination of the rate of turnover, *k*_cat_, requires knowledge of the exact enzyme concentration (*E*_0_), which is difficult to determine for UCP_PETNR_. However, the similar *K*_m_ values (Michaelis constant) for PETNR and UCP_PETNR_ suggest that conjugation of the enzyme to the UCP has not had a major effect on the enzyme activity. If one assumes that *k*_cat_ is unaffected by UCP conjugation the active enzyme concentration in the UCP_PETNR_ samples can be estimated to be ≈0.37 μg active PETNR per mg UCP (see the Supporting Information). Assuming detection is limited to the *K*_d_ for KI, the LOD for this system would be on the order of 10 μm;[^8^, ^9^] future work is focused on reducing the *K*_d_ to optimize detection limits.

As stated above, the spectral overlap between the 475 nm UC emission from PTIR‐475 and the absorption of the oxidized flavin PETNR means the emission of UCP_PETNR_ is sensitive to the oxidation state of the enzyme.[^8^, ^9^] Reduction of UCP_PETNR_ with NADPH or sodium dithionite leads to a significant increase in 475 UC emission and reoxidation with KI or molecular oxygen causes this UC emission to revert to the original value (Figures 4 and S10). The sample is stable and can be cycled multiple times between oxidized and reduced forms, demonstrating the potential of UCP‐enzyme systems for ratiometric detection of substrates, coenzymes and/or molecular oxygen.^[21]^ Incorporation of other (flavo)enzyme oxidoreductases would allow detection of a wide range of substrates and inhibitors by employing a competition assay approach.

**Figure 4:**
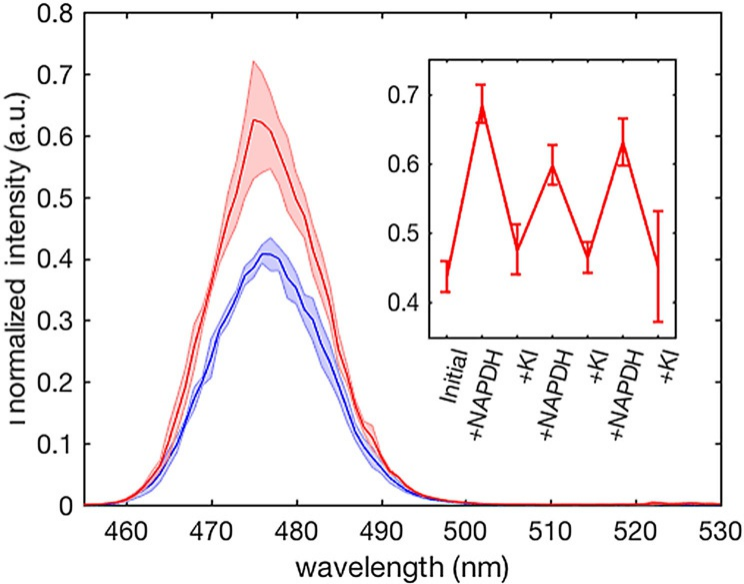
UC emission spectra of oxidized (blue) and reduced (red) UCP_PETNR_. Emission was normalized to the 800 nm emission (not shown) and the samples were excited at 980 nm. Solid lines=average, boundaries=1 standard deviation (*n=*3). The inset shows the emission of UCP_PETNR_ during repeated cycling with excess NADPH and KI. A two‐sample T‐test on variance between the means of the oxidized and reduced data points throughout the cycling experiment gave *p*=0.0008.

It should be noted that, while the scale of the UCPs largely precludes distances involved in classical energy transfer processes, it has previously been reported that the unique internal particle environment may enhance these distances and luminescent resonance energy transfer (LRET) may work much more efficiently than traditional FRET.^[22]^ We also suggest that the majority of emitter ions are excited closer to the surface of the particle, with the largest surface area and lowest penetration depth, and the potential for energy migration through the lattice from the core to the surface. In this case, energy transfer from the surface would therefore be expected to show significantly more efficiency than calculated for the bulk particle.

As the PETNR *K*_m_ for KI appears to be largely unaffected by conjugation to UCPs, (at least with these UCPs) it seems likely that sensors based on UCP‐enzymes will benefit from the inherent selectivity of native enzymes for their substrates. Sensing of redox state, oxygen levels or of specific molecules may be possible within a cellular environment using suitable enzymes functionalised to smaller, cell‐permeable UCPs and this a future directive of our research. Future incorporation of Nd^III^ into the UCPs may also allow improved sensing through access to the more biologically transparent 808 nm excitation.^[1f]^

In summary, we have covalently coupled GFP and PETNR to UCPs, with AET from the UCP to protein cofactor observed in both cases. Efficient AET requires spectral overlap of UCP emission and protein cofactor absorption, so ratiometric monitoring is possible by using a UCP emission band with no overlap with protein cofactor (e.g., the 800 nm band). PETNR remains catalytically active when coupled to the UCP and the presence of reductant or reducing substrate can be determined ratiometrically from UCP emission. This approach offers a drop‐in alternative to the use of thiol‐reactive organic fluorescent probes for use as, for example, “molecular probes”,^[17]^ while benefiting from the inherent advantages of UCP‐based detection.[^4^, ^5^]

## Conflict of interest

The authors declare no conflict of interest.

## Supporting information

As a service to our authors and readers, this journal provides supporting information supplied by the authors. Such materials are peer reviewed and may be re‐organized for online delivery, but are not copy‐edited or typeset. Technical support issues arising from supporting information (other than missing files) should be addressed to the authors.

Supplementary
