# Supplementary material for: Covalent Attachment of Active Enzymes to Upconversion Phosphors Allows Ratiometric Detection of Substrates
Source: Chemistry. 2020 Oct 16;26(65):14817–22. doi: 10.1002/chem.202001974 (PMC7756657; doi:10.1002/chem.202001974)
Supplement: Supplementary file 1 — Supplementary [file CHEM-26-14817-s001.pdf]

Chemistry–A European Journal

Supporting Information

Covalent Attachment of Active Enzymes to Upconversion Phosphors Allows Ratiometric Detection of Substrates

Letitia Burgess,[a, b] Hannah Wilson,[a, b] Alex R. Jones,[a, b, c, d] Peter Harvey,*[a, e] Louise S. Natrajan,*[a, c] and Sam Hay*[a, b]

Author Contributions

L.N. Conceptualization: Equal; Funding acquisition: Equal; Project administration: Equal; Resources: Equal; Supervision: Lead; Validation: Equal; Writing - Original Draft: Supporting; Writing - Review & Editing: Equal
S.H. Conceptualization: Equal; Formal analysis: Supporting; Funding acquisition: Equal; Project administration: Equal; Resources: Equal; Supervision: Supporting; Validation: Equal; Writing - Original Draft: Supporting; Writing - Review & Editing: Equal
L.B. Formal analysis: Lead; Investigation: Lead
H.W. Formal analysis: Supporting; Investigation: Supporting; Writing - Review & Editing: Supporting
A.J. Conceptualization: Equal; Supervision: Supporting; Validation: Supporting; Writing - Original Draft: Supporting; Writing - Review & Editing: Supporting.

TABLE OF CONTENTS

Experimental details	2
Estimation of PETNR concentration in UCP_{PETNR}	4
Scheme S-1, Detailed legend to accompany Scheme 1	4
Figure S-1, X-ray crystal structures of GFP and PETNR	5
Figure S-2, Direct fluorescence spectra of GFP in UCP_{GFP}	5
Figure S-3, Comparative UC emission spectra of UCP and UCP_{GFP}	6
Figure S-4, UC emission spectra of malUCNP and $malUCNP_{GFP}$	6
Figure S-5, UV-vis absorption spectra monitoring washing of UCP_{PETNR}	7
Figure S-6, FTIR spectra of PETNR, $UCP_{apo-PETNR}$ and UCP_{PETNR}	7
Figure S-7, TEM images of dried ^{mal}UCNP and $^{mal}UCNP_{GFP}$	8
Figure S-8, TEM images of UCP, $UCP_{apo-PETNR}$ and UCP_{PETNR}	8
Figure S-9, Dynamic light scattering (DLS) measurements	9
Figure S-10, UC emission spectra of oxidised and reduced UCP_{PETNR}.	10

EXPERIMENTAL DETAILS

Materials

The Gd_2SO_2:Yb,Tm UCPs (PTIR-475) were donated by Phosphor Technology Ltd. (https://www.phosphor-technology.com/laser-detection-phosphors/). Sulfo-SMCC was purchased from Thermo-Fischer Scientific. All other solvents and reagents were purchased from Sigma-Aldrich. All reagents and solvents were used as received.
Deionised (DI) water was obtained from a Millipore Synergy water purification system.

Protein preparation

PETNR with a C-terminal His_8 tag was prepared as described previously (H. S. Toogood, A. Fryszkowska, M. Hulley, M. Sakuma, D. Mansell, G. M. Stephens, J. M. Gardiner and N. S. Scrutton, *ChemBioChem*, 2011, **12**, 738-749).
EGFP with an N-terminal His_6 tag was expressed in *E. coli* BL21 DE3 and purified by nickel affinity chromatography.

SYNTHETIC DETAILS

Synthesis of 3-aminopropyltriethyoxysilane Gd_2SO_2:YbTm (UCP_{APTES})

Gd_2SO_2:YbTm (26 mg) were dispersed in a mixture of Igepal CO-520 (4mL, 9.04mmol) in cyclohexane (80 mL) before 25 % ammonium hydroxide was added (w/w, 900 uL, 22.6 mmol). After sonication for 30 minutes, TEOS (200 µL, 0.9 mmol) was added and the reaction stirred for 60 minutes. APTES (200 µL, 0.85 mmol) was then added and the reaction mixture left to stir at room temperature for 48 hours in a sealed flask. Acetone (40 mL) was added to cause the nanoparticles to precipitate. Collected by centrifugation (4000 rpm, 10 minutes) and washed twice with acetone (30 mL) and twice with EtOH:H_2O (2:1, 30 mL).

Covalent attachment of Green Fluorescent Protein (GFP) to Gd_2SO_2:YbTm (UCP_{GFP})

Sulfo-SMCC (1 mg, 0.0023 mmol) was dissolved in deionised water (100 µL) then diluted with PBS (500 µL, pH 7.4, 100 mM). GFP (40 µmol) was added and the reaction mixture was left to react at room temperature for 30 minutes before the addition of APTES475 (52.1 mg, aqueous solution in 1 mL H_2O). The reaction was left to react at 4 ^{o}C under gentle agitation for 24 hours. The product was collected by centrifugation (10 minutes, 10000 rpm) and washed three times with PBS (1 mL, pH 7.4, 100 mM) until the supernatants were shown to be free of unbound GFP by UV-Vis spectroscopy. The concentration of loaded GFP per mg of UCP_{GFP} was determined by solution UV-Vis spectroscopy of the final product (1mg mL^{-1}) using the experimentally determined molar absorption extinction coefficient that matched that reported in the literature[1] and the number of moles GFP per mg nanoparticle calculated as 3.9 nanomoles of GFP per 1 mg of UCP_{GFP}, resulting in a working concentration of 3.9 µM GFP in the 1mg mL^{-1} UCP solutions.

Synthesis of maleimide-capped nano-UCPs (^{mal}UCNP)

6-malimidohexanoic acid 11.4 g, 54 mmol) was dissolved in ethanol (25 mL) then added to a 4 mL aqueous solution of NaOH (1 g, 24.4 mmol) and stirred for 10 mins. 1.2 mL $LnCl_3$ (0.5 M, 80 % Y^{III}, 20 % Yb^{III}, 0.2 % Tm^{III}) was added and stirred for another 10 minutes, before 4 mL aqueous NaF (1 M, 0.168 g) was added dropwise. The mixture was stirred for 10 mins then placed in a Teflon lined reaction vessel and heated at 120 °C for 30 minutes before increasing the temperature to 200 °C for 5 hours. The solution was centrifuged after cooling and washed with ethanol (3 x 10 mL).

Covalent attachment of GFP to ^{mal}UCNP (^{mal}UCNP$_{GFP}$)
^{mal}UCNP$_{GFP}$ (18.6 mg) was suspended in n 1 mL H_2O then diluted with PBS (1 mL, pH 7.4, 100 mM). GFP (13 µM) was then added before the reaction was left to react at 4 °C under gentle agitation for 24 hours. The product was collected by centrifugation (10 minutes, 10,000 rpm) and washed three times with PBS (3 x 1 mL, pH 7.4, 100 mM).

Covalent attachment of Pentaerythritol Tetranitrate Reductase to Gd_2SO_2:YbTm (UCP$_{apo\text{-}PETNR}$)
Sulfo-SMCC (2 mg, 0.0046 mmol) was dissolved in deionized water (200 µL) then diluted with PBS (1 mL, pH 7.4, 100 mM). PETNR (145 µM) was added and the reaction mixture was left to react at room temperature for 30 minutes before the addition of APTES475 (10 mg, aqueous solution in 1 mL H_2O). The reaction was left to react at 4 ^{o}C under gentle agitation for 24 hours. The product was collected by centrifugation (10 minutes, 10000 rpm) and washed three times with PBS (1 mL, pH 7.4, 100 mM) until the supernatants were shown to be free of unbound PETNR by UV-visible spectroscopy.

Reintroduction of flavin mononucleotide into PETNR475 (UCP$_{PETNR}$)
UCP$_{apo\text{-}PETNR}$ (5 mg) were suspended in PBS (3 mL, pH7.4, 100 mM), added KBr (0.119 g, 1 mmol) and FMN (50 mg, 0.11 mmol). Reaction mixture left to react for 24 hours at 4 ^{o}C under gentle agitation. Collected the product by centrifugation (10 minutes, 4000 rpm) and washed four times with PBS (10 mL, pH 7.4, 100 mM) until the supernatants were shown to be free of unbound FMN by UV-Visible spectroscopy.

Nanoparticle characterisation
The TEM images were obtained on a 200 kV Phillips Microscope on carbon coated copper grids and were analysed using the Gatan3 digital micrograph software.
Infrared spectra were obtained using solid samples on a Bruker alpha FT-IR spectrometer, data was analysed using Origin.
DLS size and zeta potential data was recorded in disposable folded capillary cells on a Malvern Zetasizer nano Z instrument.
UV-visible spectra were recorded on a Cary 60 Spectrophotometer (Aglient) using a 1 cm^3 quartz cuvette, data was analysed using Origin. All UCP solutions were 1 mg mL^{-1}.

Luminescence Spectroscopy Measurements
Luminescence spectra were recorded on an Edinburgh Instrument FP920 Phosphorescence Lifetime Spectrophotometer equipped with a red sensitive photomultiplier in peltier (air cooled) housing, (Hamamatsu R928P) using a 1 cm^3 quartz cuvette and a 45 mW 980 nm diode laser operating on either continuous wave or pulsed mode, all spectra were recorded uncorrected in order for the 475 nm band to be visible and the data was analysed using Origin. The final data was normalized to the 800 nm band in order to negate probe concentration effects. Lifetime data were recorded after excitation using the 45 mW 980 nm diode laser on pulsed mode using time correlated single photon counting (PCS900 plug-in PC card), and the data was fitted using the exponential tail fit data processing package in the Edinburgh Instrument software program. All UCP solutions were 1 mg mL^{-1}.

Enzyme Kinetic Studies
Steady state kinetic assays were carried out for PETNR (100 nM) and PETNR(FMN)475 (1 mg mL^{-1}) after addition of NADPH (100 µM) and varying concentrations of ketoisophoron. Reactions were performed in 100 mM Tris, pH 7 under anaerobic conditions. The reaction was monitored

by following the loss in absorbance at 340 nm associated with reduced NADPH (ε = 6.22 mM^{-1} cm^{-1}).

Reduction/Oxidation Experiments

Reduction of the enzyme bound to PETNR(FMN)475 (1 mg/mL in degassed, 100 mM TRIS, pH 7) was carried out in a 1 cm^3 quartz cuvette by addition of a reducing agent (200 μM NADPH or 400 μM sodium dithionite (SD)). The Oxidised enzyme was re-generated either by addition of the ketoisophorone (120 μM) or by exposing the system to atmospheric oxygen. Emission from the UCNPs was recorded following excitation with a 45 mW CW 980 nm diode laser.

Estimation of PETNR concentration in UCP_{PETNR}

$k_{cat} = \frac{V_{max}}{[E_0]}$ rearranges to give $E_0 = \frac{V_{max}}{k_{cat}}$

$$E_0 = \frac{0.04187\ \mu\mathrm{M}\ s^{-1}}{4.51\ s^{-1}} = 0.0093\frac{\mu M}{\frac{mg}{mL}} = 0.0093\ nmol\ mg^{-1}$$

The molecular weight of PETNR is 40 kDa, therefore:

$$E_0 = 40000\ ng\ nmol^{-1}\ x\ 0.0093\ nmol\ mg^{-1} = 373.3\ ng\ mg^{-1} = 0.373\ \mu g\ mg^{-1}$$

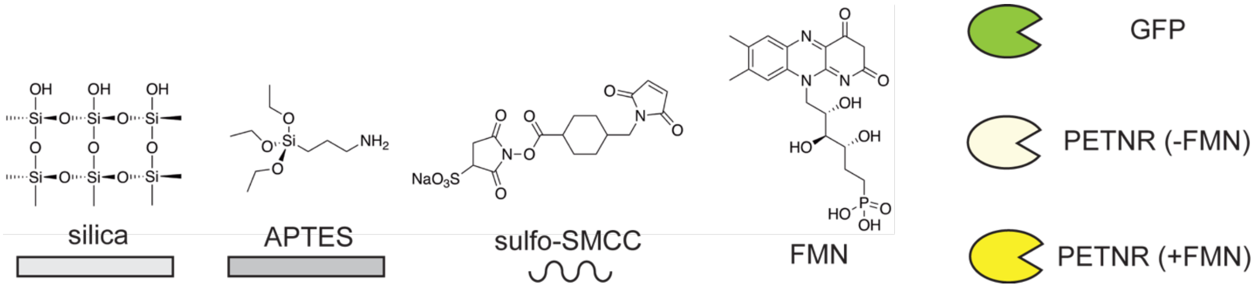

Scheme S-1 Detailed legend to accompany Scheme 1 in the main text.

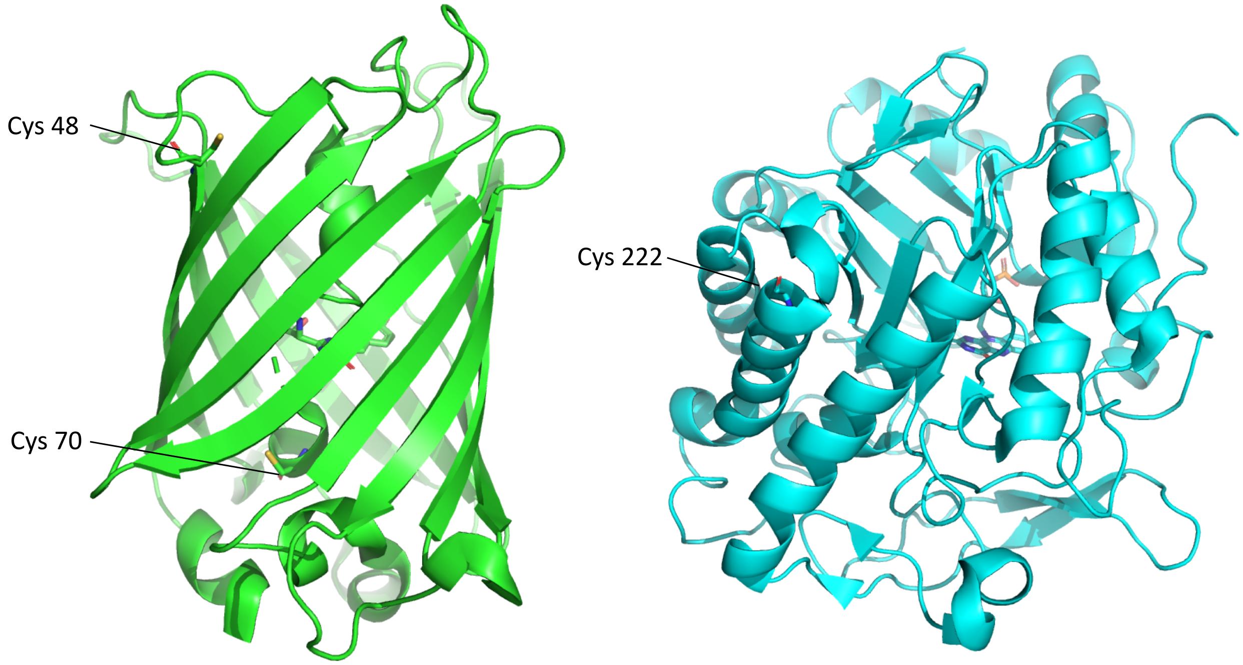

Figure S-1 X-ray crystal structures of enhanced GFP (green; PDB ID: 2Y0G) and His-tagged PETNR (teal; PDB ID: 3P62) with cysteine residues labelled.

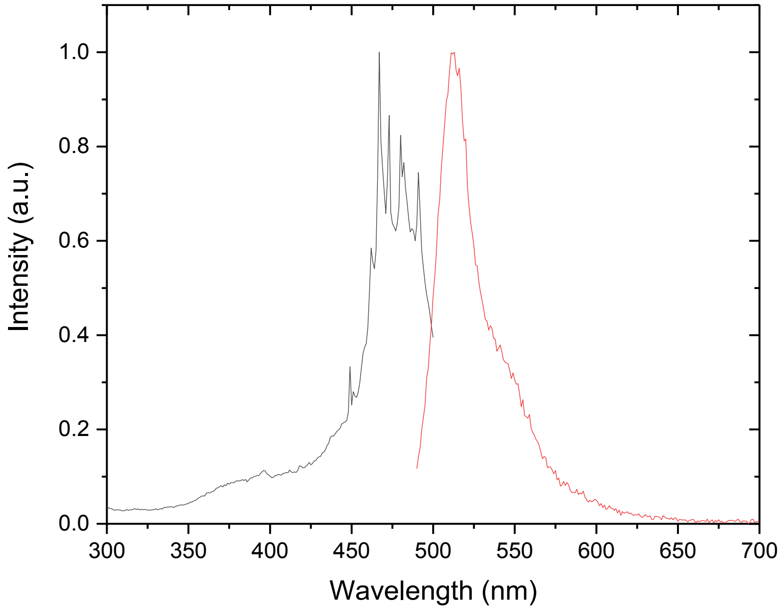

Figure S-2 Direct excitation (black) and emission (red) fluorescence spectra of GFP in UCP_{GFP}. As is the case for PETNR (Figure 2 in the main manuscript), the fine structure in the excitation spectra is likely due to emission from Tm^{III} in the UCPs.

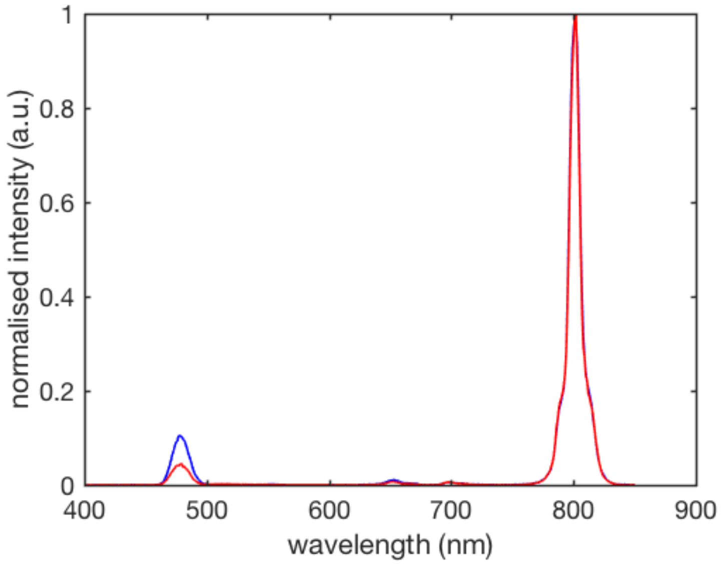

Figure S-3 Comparative upconversion emission spectra of UCP (blue) and UCP_{GFP} (red) in phosphate buffered saline (PBS) (λ_{ex} = 980 nm), with expected decrease in 475 nm emission due to energy transfer to GFP. Bands are normalized to the 800 nm UC emission intensity, and expanded 475 nm spectra are shown in Figure 1D of the main manuscript.

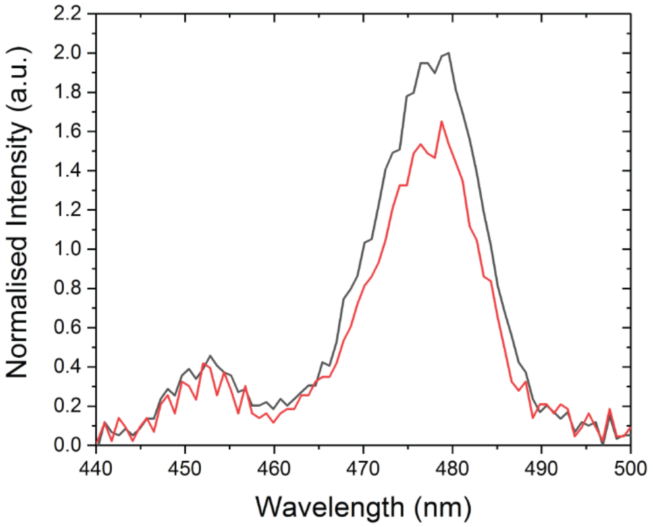

Figure S-4 UC emission spectra of synthesised ^{mal}UCPs before (^{mal}UCNP, black) and after ($^{mal}UCNP_{GFP}$, red) covalent conjugation of GFP to maleimide-capped nano-UCPs. λ_{ex} = 980 nm and spectra are normalized to the 800 nm UC emission intensity.

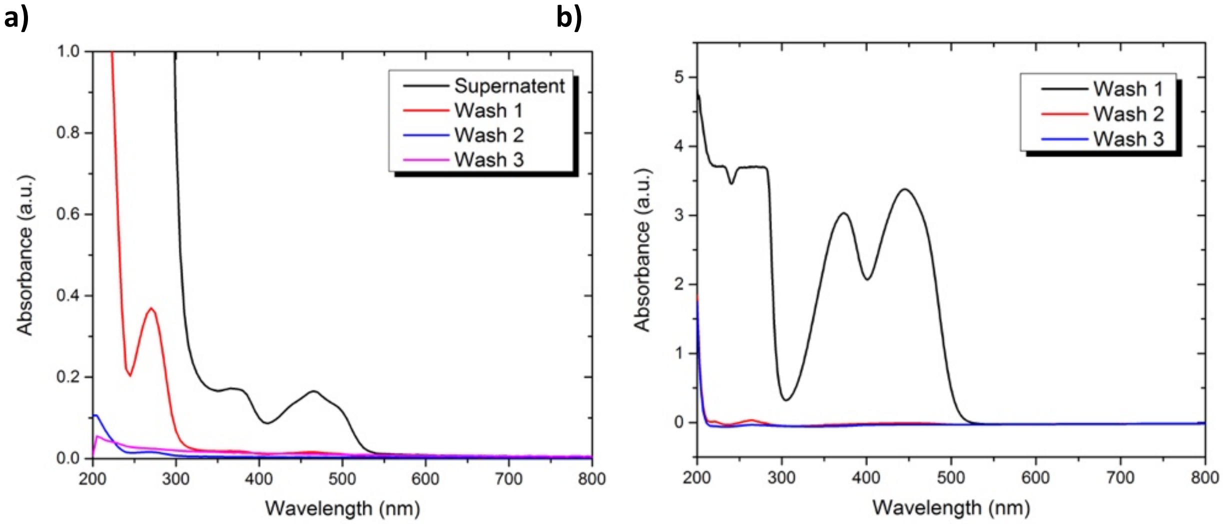

Figure S-5 UV-vis absorption spectra of **(a)** the supernatant obtained after each wash of $^{FMN-}$UCP$_{PETNR}$ after covalent attachment of PETNR (100 mM tris, pH 7) and **(b)** the supernatant obtained after each wash of the UCP$_{PETNR}$ after the reintroduction of FMN (100 mM PBS, pH 7.47).

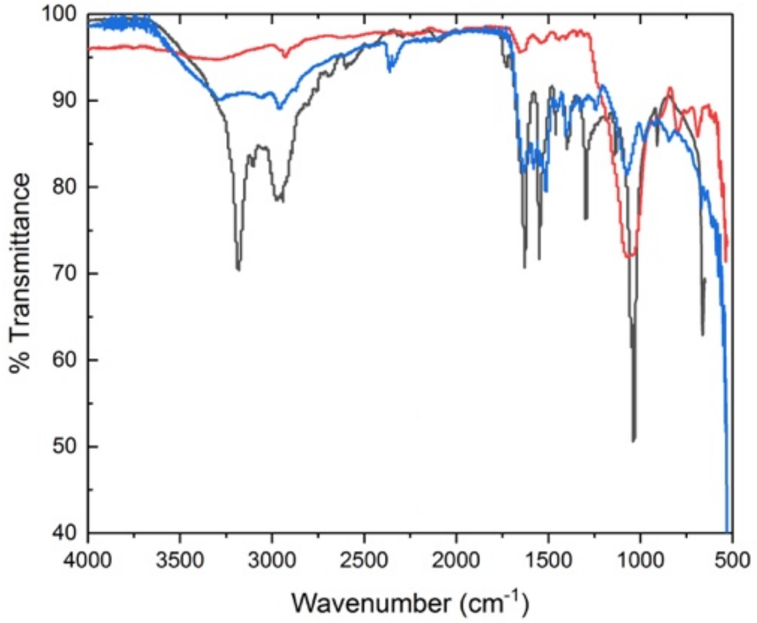

Figure S-6 Fourier-transform infrared (FTIR) spectra of PETNR (blue), UCP$_{apo-PETNR}$ (red), and UCP$_{PETNR}$ (black). All spectra were recorded as dried solid samples, the Raman was obtained using a 532 nm laser. Stretching frequencies at 3220 cm^{-1} (O-H), 2940 (C-H), 1620 cm^{-1} (C=O), 1510 cm^{-1} (N-H), and 1040 cm^{-1} (Si-O-Si) signify silica-coating and the presence of organic matter (protein).

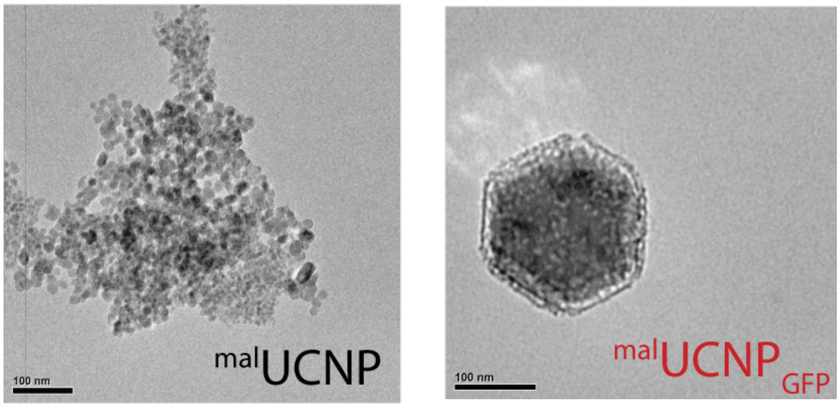

Figure S-7 Representative transmission electron microscopy (TEM) images displaying distinct change in morphology of dried ^{mal}UCNP samples upon binding of GFP. Scale bars 100 nm.

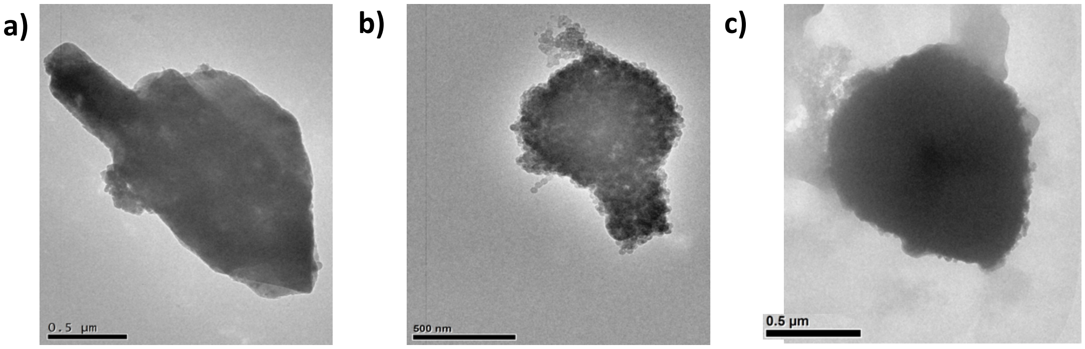

Figure S-8 Representative transmission electron microscopy (TEM) images of **(a)** UCP, **(b)** $UCP_{apo\text{-}PETNR}$ and **(c)** UCP_{PETNR}. Samples were prepared by drop casting a 1mg/mL UCP suspension onto 200 mesh copper grids with carbon mesh. Scale bars 500 nm. Average sizes were determined from at least 10 measurements and are determined as 765 nm (UCP), 809 nm (UCP_{APTES}), 990 nm ($UCP_{apo\text{-}PETNR}$) and 939 nm (UCP_{PETNR}).

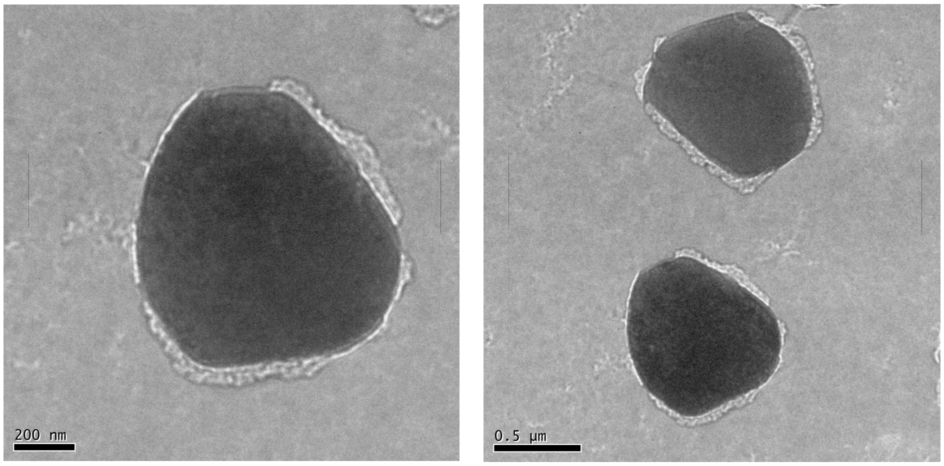

Figure S-9 Representative transmission electron microscopy (TEM) images of UCP_{GFP}. Samples were prepared by drop casting a 1mg/mL UCP suspension onto 200 mesh copper grids with carbon mesh. Scale bars 200 and 500 nm. The average size was determined as 878 nm.

Table S-1 Dynamic light scattering (DLS) measurements (1mg/mL in 100 mM TRIS pH 7). Note that there is a high degree of uncertainty in the measurements due to sedimentation of the UCPs from the solution.

**Sample**	**Diameter (nm)**	**PDI**	**Zeta Potential (mv)**
UCP	1432	0.4	3.2
UCP_{APTES}	4964	0.8	-11.1
$UCP_{apo\text{-}PETNR}$	13590	1.0	-14.2
UCP_{PETNR}	794	0.6	-20.1
UCP_{GFP}	1183	0.4	-4.0

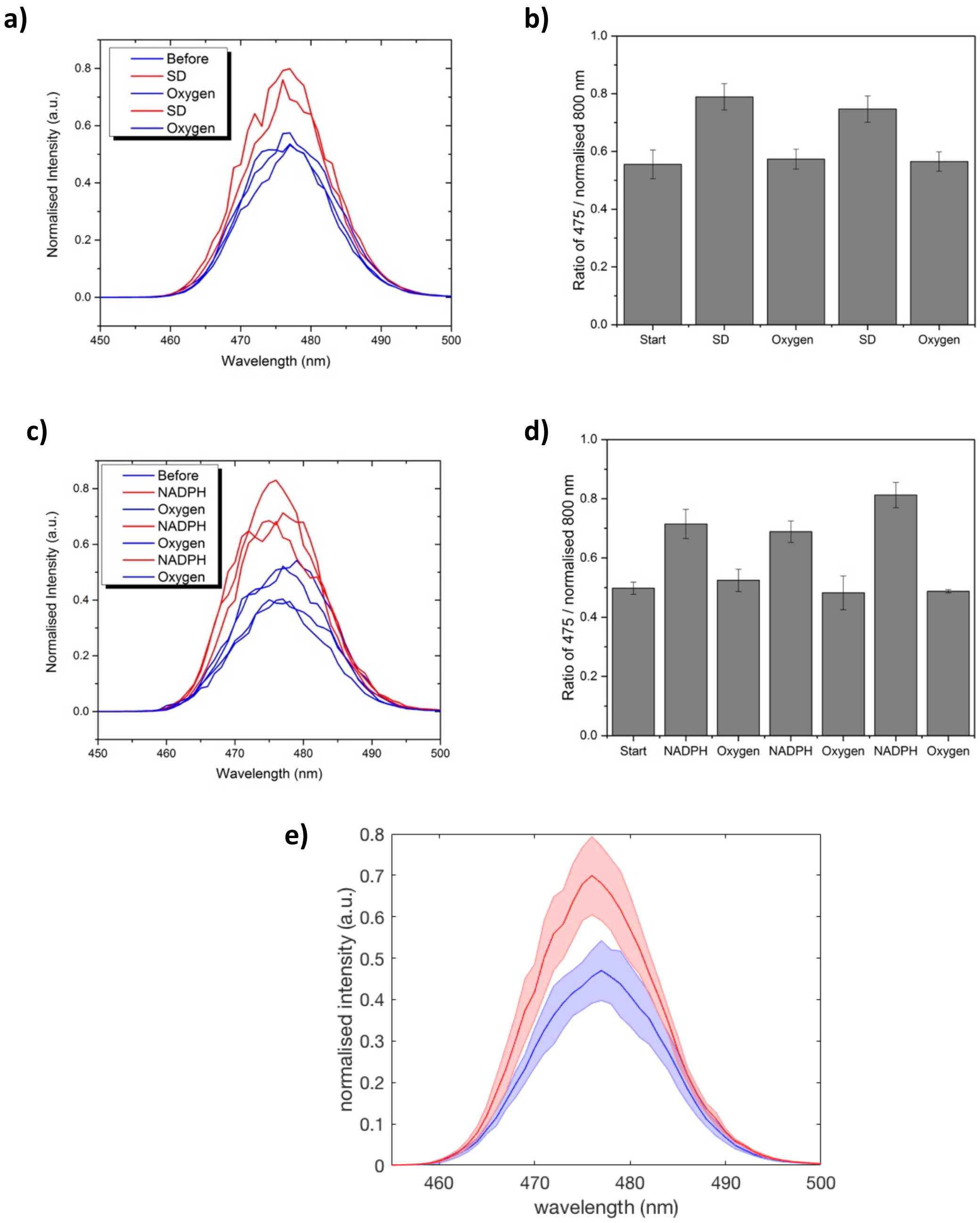

Figure S-10 (a & c) Upconversion emission spectra of oxidised (blue) and reduced (red) UCP_{PETNR}. Individual plots represent repeated cycling between the two states as represented in the bar charts **(b & d)**. Samples were alternately reduced with either sodium dithionite (SD, **a & b**) or reduced nicotinamide adenine dinucleotide phosphate (NAPDH, **c & d**) then oxidised by environmental oxygen. The comparative data for cycling with NADPH and O_2 is shown in Figure 3 in the main manuscript. **(e)** Compilation of all oxidised and reduced data in Figures 3, S-8a and S-8c, showing clear distinction between the two states. Solid lines = average, blurred boundaries = one standard deviation.

References:

1. S.R. McRae, C.L. Brown, G.R. Bushell, *Protein Expres Purif.* **2005**, *41*, 121-127.
